# Improved chromosome-level genome assembly and annotation of the seagrass, *Zostera marina* (eelgrass)

**DOI:** 10.12688/f1000research.38156.1

**Published:** 2021-04-15

**Authors:** Xiao Ma, Jeanine L. Olsen, Thorsten B.H. Reusch, Gabriele Procaccini, Dave Kudrna, Melissa Williams, Jane Grimwood, Shanmugam Rajasekar, Jerry Jenkins, Jeremy Schmutz, Yves Van de Peer

**Affiliations:** 1 Department of Plant Biotechnology and Bioinformatics, Ghent University - Center for Plant Systems Biology, VIB, Ghent, 9052, Belgium; 2 Groningen Institute of Evolutionary Life Sciences, Groningen, 9747 AG, The Netherlands; 3 GEOMAR Helmholtz Centre for Ocean Research Kiel, Marine Evolutionary Ecology, Kiel, 24105, Germany; 4 Department of Integrative Marine Ecology, Stazione Zoologica Anton Dohrn, Napoli, 80123, Italy; 5 Department of Energy Joint Genome Institute, Lawrence Berkeley National Lab, Berkeley, CA, USA; 6 HudsonAlpha Institute for Biotechnology, Huntsville, AL, USA; 7 Arizona Genomics Institute, School of Plant Sciences, University of Arizona Tucson, Tucson, AZ, 85721, USA; 8 Department of Biochemistry, Genetics and Microbiology, University of Pretoria, Pretoria, South Africa; 9 College of Horticulture, Nanjing Agricultural University, Nanjing, 210014, China

**Keywords:** Seagrass, Zostera marina, eelgrass, chromosome-scale genome assembly, annotation

## Abstract

**Background:** Seagrasses (Alismatales) are the only fully marine angiosperms. *Zostera marina *(eelgrass) plays a crucial role in the functioning of coastal marine ecosystems and global carbon sequestration. It is the most widely studied seagrass and has become a marine model system for exploring adaptation under rapid climate change. The original draft genome (v.1.0) of the seagrass *Z*. *marina* (L.) was based on a combination of Illumina mate-pair libraries and fosmid-ends. A total of 25.55 Gb of Illumina and 0.14 Gb of Sanger sequence was obtained representing 47.7× genomic coverage. The assembly resulted in ~2000 unordered scaffolds (L50 of 486 Kb), a final genome assembly size of 203MB, 20,450 protein coding genes and 63% TE content. Here, we present an upgraded chromosome-scale genome assembly and compare v.1.0 and the new v.3.1, reconfirming previous results from Olsen et al. (2016), as well as pointing out new findings.

**Methods:** The same high molecular weight DNA used in the original sequencing of the Finnish clone was used. A high-quality reference genome was assembled with the MECAT assembly pipeline combining PacBio long-read sequencing and Hi-C scaffolding.

**Results:** In total, 75.97 Gb PacBio data was produced. The final assembly comprises six pseudo-chromosomes and 304 unanchored scaffolds with a total length of 260.5Mb and an N50 of 34.6 MB, showing high contiguity and few gaps (~0.5%). 21,483 protein-encoding genes are annotated in this assembly, of which 20,665 (96.2%) obtained at least one functional assignment based on similarity to known proteins.

**Conclusions:** As an important marine angiosperm, the improved *Z. marina* genome assembly will further assist evolutionary, ecological, and comparative genomics at the chromosome level. The new genome assembly will further our understanding into the structural and physiological adaptations from land to marine life.

## Introduction

Seagrasses are a polyphyletic assemblage of early-diverging monocotyledoneous plants belonging to the Alismatales ( Les, Cleland, and Waycott 1997; Du and Wang 2016); they are not true grasses (Poaceae). Several clades of seagrasses arose independently from freshwater sister taxa 3-4 times between the Paleocene and late Eocene (~65-34 mya) and are the only fully marine, flowering plants (~14 genera and ~65 species) ( Chase et al. 2016). They occur in predominantly soft-sediment, marine coastal environments worldwide ( Green, Short, and Frederick 2003) and as engineering species provide the foundation of three-dimensional habitats that are among the most productive and biodiverse ( Costanza et al. 1997; Duffy et al. 2015). Seagrass meadows provide numerous ecosystem services, e.g., provisioning of fish and invertebrates, retention of nutrients ( Larkum, Orth, and Duarte 2006) and carbon sequestration ( Fourqurean et al. 2012). Unfortunately, they are also under threat related to human impacts ( Waycott et al. 2009) that fundamentally change coastal system dynamics ( Duffy et al. 2015) and make restoration extremely difficult ( van Katwijk et al. 2016). Effective marine conservation strategies require integrative research perspectives between ecology and genomics ( Hillebrand, Jacob, and Leslie 2020) because ecological and evolutionary change can and do occur on the same time scales ( Carroll et al. 2007), e.g., genetic polymorphisms underlying critical traits or the role of genetic diversity at selectively relevant sites for population resilience.

*Zostera marina* (eelgrass) is a marine model species with >3000 papers covering a wide variety of ecological, evolutionary, conservation and biotech topics. Its unique, circumglobal, warm temperate to Arctic distribution has allowed it to withstand numerous cycles of rapid climate change during the Pliocene glacial and interglacial periods ( Olsen et al. 2004), empirically demonstrating its capacity to adapt, acclimate and recover ( Duarte et al. 2018), e.g., to temperature ( Franssen et al. 2011; Jueterbock et al. 2016; Jueterbock et al. 2020), salinity gradients/osmoregulation ( Shivaraj et al. 2017), ocean acidification ( Zimmerman 2020) and potential pathogens ( Brakel et al. 2014; Guan, Saha, and Weinberger 2020; Zang et al. 2020). Further, clonal populations of *Z. marina* can persist for hundreds to thousands of years (( Thorsten et al. 1999) for *Z. marina*, ( Arnaud-Haond et al. 2012) for *Posidonia oceanica*)), yet have found ways to adapt through periods of rapid climate selection via genotypic plasticity, fostered by somatic mutation ( Yu et al. 2020) and epigenetic modification of the methylome ( DuBois, Williams, and Stachowicz 2020; Jueterbock et al. 2020). Microbiome interaction studies are being conducted in parallel with eelgrass resequencing, e.g., bacterial ( Cucio et al. 2016; Fahimipour et al. 2017; Wang, Tomas, and Mueller 2020; Eisen et al. 2017) and fungal ( Ettinger, Voerman, et al. 2017; Ettinger, Williams, et al. 2017; Ettinger and Eisen 2019, 2020; Ettinger, Vann, and Eisen 2020) to inform restoration strategies as well as meta-organismal function. Bioengineered salinity tolerance is also of interest ( Wani et al. 2020).

The new assembly of the *Z. marina* reference genome will further advance studies in the aforementioned areas, as well as comparative analyses of genome structure and evolution, as new reference genomes for representatives of the other three seagrass lineages (i.e., *Posidonia oceanica - Posidoniaceace*, *Cymodocea nodosa - Cymodoceaceae* and *Thalassia testudinum - Hydrocharitaceae*) come online in the near future along with *Zostera mueller* ( Lee et al. 2016) and *Zostera japonica* (unpublished).

## Methods

### Sequencing strategy

We used an aliquot of the same DNA that served as the basis for *Z. marina* v.1.0 genome. We sequenced the *Z. marina* genome using a whole genome shotgun sequencing strategy and standard sequencing protocols. Sequencing reads were collected using Illumina and PacBio platforms at the HudsonAlpha Institute for Biotechnology in Huntsville, Alabama, USA. Illumina reads were sequenced using the Illumina HiSeq-2500 platform and the PacBio reads were sequenced using the SEQUEL II platform. One 400 bp insert 2×150 Illumina fragment library (162.7× coverage), and one 2×150 Hi-C Illumina library were constructed using Dovetail Hi-C kit and sequenced to 581.1× coverage. Prior to assembly, Illumina fragment reads were screened for PhiX contamination. Reads composed of >95% simple sequence were removed. Illumina reads <50 bp after trimming for adapter and quality (q<20) were removed. The final read set consists of 280,181,449 reads for a total of 156.4× of high-quality Illumina bases. For the PacBio sequencing, a standard PacBio long read library was constructed and a total of 8 PB chemistry 3.0 chips (10 hours movie time) were sequenced on a Sequel 1, a sequence yield of 75.97 Gb, with a total coverage of 189.93x.

### Genome assembly and construction of pseudomolecule chromosomes

The current v.3.1 assembly was generated by assembling the 5,615,408 PacBio reads (189.93x sequence coverage) using the MECAT assembler ( Xiao et al. 2017) and subsequently polished using ARROW ( Chin et al. 2013).

Misjoins in the assembly were identified using Hi-C data as part of the JUICER pipeline ( Durand et al. 2016). No misjoins were identified in the polished assembly. The contigs were then oriented, ordered, and joined together using HI-C data using the JUICER pipeline. A total of 89 joins were applied to the assembled contigs to form the final set of six chromosomes. Each chromosome join is padded with 10,000 Ns. Significant telomeric sequence was identified using the (TTAGGG) _n_ repeat, and care was taken to make sure that it was properly oriented in the production assembly. The remaining scaffolds were screened against bacterial proteins, organelle sequences, GenBank nr and removed if found to be a contaminant.

Finally, homozygous SNPs and INDELs were corrected in the released consensus sequence using 40x of Illumina reads (2x150, 400bp insert) by aligning the reads using bwa mem ( Li 2013) and identifying homozygous SNPs and INDELs with the GATK’s UnifiedGenotyper tool ( McKenna et al. 2010). A total of 1,876 homozygous SNPs and 64,447 homozygous INDELs were corrected in the release.

### Annotation of repetitive elements and noncoding RNAs

RepeatModeler v2.0 was used to build a custom repeat library for the genome assembly of *Z. marina* v.3.1. Subsequently, RepeatMasker v4.1 was used to discover and classify repeats based on the custom repeat libraries from RepeatModeler v2.0. Transfer RNAs (tRNA) were predicted by tRNAscan-SE v1.31 ( Chan and Lowe 2019) with default parameters. We also identified noncoding RNAs, such as microRNAs (miRNAs), small nuclear RNAs (snRNAs) and ribosomal RNAs (rRNAs) by comparing with known noncoding RNA libraries (Rfam v14.2), using the cmscan program of Infernal v1.1.2 ( Nawrocki and Eddy 2013). In addition, novel miRNA entries from the *Z. marina* v.1.0 assembly were aligned to hard-masking *Z. marina* v.3.1 using SeqMap ( Jiang and Wong 2008) with no mismatches. We extracted ~ 110 bp upstream and downstream sequences surrounding every aligned locus and discarded the miRNAs candidates located within protein coding sequences or repetitive elements (“NNNNNNNNNNN”). The stem-loop structure and the minimum free energy (MFE) were predicted for each region using the RNAfold program of the ViennaRNA v 2.1.1 ( Lorenz et al. 2011) with default settings. Finally, the results based on Rfam and *Z.marina* v.1.0 were combined into a non-redundant prediction of miRNAs.

### Gene prediction and functional annotation

Genome annotation was performed using a combination of *ab initio* prediction, homology searches and RNA-aided alignment. Augustus ( Stanke, Tzvetkova, and Morgenstern 2006) was used for *ab initio* gene prediction using model training based on protein structures and RNA-seq data from *Z. marina* v.1.0 ( Olsen et al. 2016). For homology-based predictions, the protein sequences of *Z. marina* v.1.0 and *Oryza sativa* were downloaded from PLAZA ( https://bioinformatics.psb.ugent.be/plaza/) and aligned to *Z. marina* v.3.1 using TBLASTN with different e-values ( *Z. marina* v.1.0 with e-value ≤ 1e-10 and *O. sativa* with e-value ≤ 1e-5). Next, regions were mapped by these query sequences to define gene models using Exonerate ( Slater and Birney 2005). For RNA-aided annotation, we downloaded 23 libraries of *Z. marina* v.1.0 from NCBI (BioProject PRJNA280336). Firstly, we joined the paired-end reads using clc_assembly_cell to generate almost 2/3 of joined reads and 1/3 of un-joined reads. Then, we aligned the joined and un-joined RNA-seq data to *Z. marina* v.3.1 using HISAT2 ( Kim, Langmead, and Salzberg 2015) with the parameters “--max-intronlen 50000” and assembled into potential transcripts using StringTie v2.1.1 ( Kovaka et al. 2019). Further, TransDecoder v5.0.2 was used to predict open reading frames (ORFs) within the assembled transcripts. Finally, gene models obtained from all three approaches were integrated as the final non-redundant gene set using EVidenceModeler (v.1.1.1) ( Haas et al. 2008). Specifically, a set of 1,460 bad gene models identified by the wgd software ( Zwaenepoel and Van de Peer 2019) was manually curated using the genome browser GenomeView on the ORCAE platform ( https://bioinformatics.psb.ugent.be/orcae/) and the gene annotation results were evaluated by BUSCO hits. Putative gene function was determined using InterProScan ( Jones et al. 2014) with the different databases, including PFAM, Gene3D, PANTHER, CDD, SUPERFAMILY, ProSite, and GO. Meanwhile, functional annotation of predicted genes was also obtained by aligning the protein sequences against the sequences in public protein databases (Uniprot/SwissProt database) using BLASTP with an e-value cut-off of 1×10-5.

## Results and discussion

### Genome size and assembly

A single genotype (or clone) of *Z. marina* from the northern Baltic Sea, Finnish Archipelago Sea had been subjected to whole-genome assembly using Sanger and Illumina sequencing (referred to as *Z. marina* v.1.0) ( Olsen et al. 2016). Since PacBio technology can deliver longer reads, necessary to improve assembly contiguity and obtain a nearly complete, reference genome, we re-sequenced the inbred, Finnish clone, leading to the final v.3.1 release, which contains 260.5 Mb of sequence, consists of 432 contigs with a contig N50 of 7.0 Mb and a total of 87.6% of assembled bases into six pseudo-chromosomes (2n = 12). Interestingly, during the assembly of the genome using Hi-C, it was noted that there was a seventh “chromosome” (scaffold_7 in this release) with a length of 8.68Mb, consisting of mainly repetitive DNA and a possible Nucleolus Organizing Region (NOR). Completeness of the euchromatic portion of v.3.1 assembly was assessed using 20,450 annotated genes from *Z. marina* v.1.0. The screened alignments indicate that 20,342 (99.47%) of the previously annotated version 1.0 genes aligned to the v.3.1 release. Of the remaining genes, 50 (0.24%) aligned at <50% coverage, while 58 (0.29%) were not found in the v.1.0 release. This shows a high degree of consistency between the two versions. However, version 3.1 presents much higher contiguity and fewer gaps compared to the previously published *Z. marina* v.1.0 ( Table 1).

**Table 1.** Comparison of genome assemblies for *Zostera marina* v1.0 ( Olsen et al., 2016) and v3.1 (current study).

Parameters	*Zostera marina* v1.0	*Zostera marina* v3.1
Scaffolds length (Mb)	203	260.5
Scaffolds number	2,228	310
Scaffolds N50 (Mb)	0.486	34.6
Contigs length (Mb)	191	259.3
Contigs number	12,583	432
Contigs N50 (Mb)	0.08	7
Largest length (bp)	2,654,544	42,612,672

### Repetitive elements and noncoding RNAs

We used *ab initio* approaches to identify and annotate repetitive sequences, which accounted for 67.12 % of *Z. marina* v.3.1. 41.72 % of these TEs were long terminal repeat (LTR) elements ( Table 2). Screening the *Z. marina* v.3.1 genome against the Rfam v14.2 database using Infernal identified 546 tRNAs, 376 rRNAs, 93 miRNAs, and 134 snRNAs ( Table 3). In addition to the 93 known conserved miRNAs identified from Rfam v14.2, we also identified 23 novel miRNAs candidates compared to 19 novel miRNAs candidates in *Z. marina* v.1.0, resulting in a total of 116 miRNAs ( Table 4).

**Table 2.** Annotation of repeat elements.

		*Zostera marina* v.3.1	
**Repeat types**		**Length (bp)**	**P (%)**
DNA		14,881,010	5.71
LINE		10,538,203	4.05
SINE		228,565	0.09
LTR	Gypsy	83,650,636	32.11
	Copia	24,717,775	9.49
	Pao	309,173	0.12
Other		4,041,971	1.55
Unknown		36,466,934	14.00
Total		174,834,267	67.12

**Table 3.** Known miRNAs identified in the genome of *Zostera marina* (v1.0 and v3.1).

	Number of loci	
miRNA family	*Zostera marina* v1.0	*Zostera marina* v3.1
miR1388	0	11
miR156	5	7
miR159	1	4
miR160	3	3
miR164	3	4
miR166	4	5
miR167	2	2
miR168	1	1
miR169	2	6
miR171	3	13
miR172	1	3
miR390	2	2
miR393	2	2
miR396	2	4
miR398	0	1
miR399	2	5
miR528	1	5
miR4414	1	8
miR5658	1	7
**Total**	36	93

**Table 4.** Novel miRNA candidates in *Zostera marina* (v1.0 and v3.1).

	Number of loci	
Novel miRNA family	*Zostera marina* v1.0	*Zostera marina* v3.1
zmr-miR001	2	2
zmr-miR002	1	0
zmr-miR003	1	1
zmr-miR004	1	1
zmr-miR005	1	1
zmr-miR006	1	3
zmr-miR007	1	1
zmr-miR008	1	0
zmr-miR009	1	3
zmr-miR010	1	1
zmr-miR011	1	1
zmr-miR012	1	1
zmr-miR013	3	7
zmr-miR014	1	1
zmr-miR015	2	0
**Total**	19	23

### Protein-coding genes

**Figure 1.**
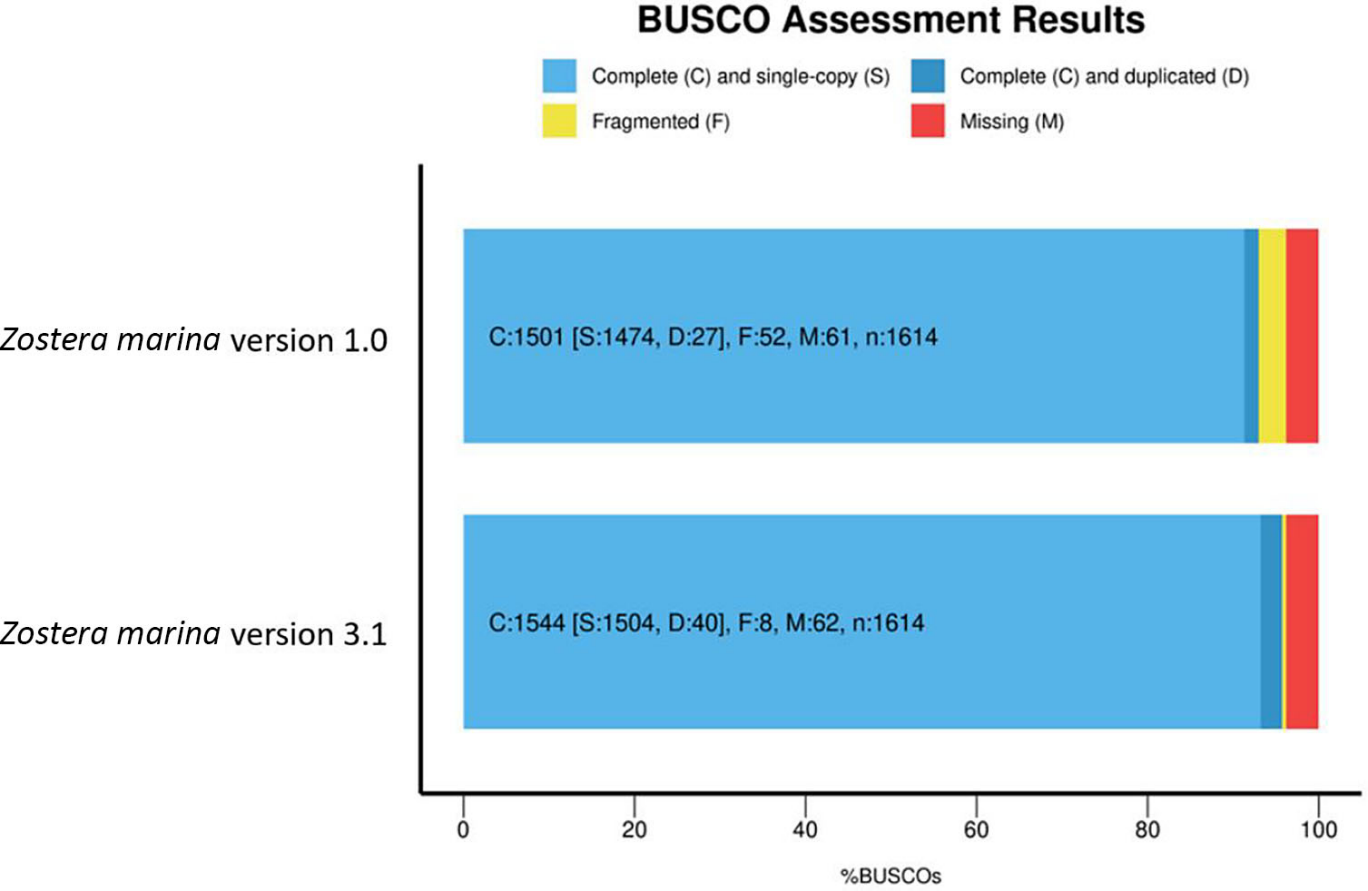
Busco assessment results for *Z. marina* genomes v1.0 and v3.1.

Through a combination of *ab initio* prediction, homology searches and RNA-aided evidence, we annotated 21,533 gene models after masking repeat elements. After manually checking most gene models and improving 1395 incorrect gene models on the ORCAE platform and adding 30 new genes based on RNA-seq evidence, the final annotation produced 21,483 gene models, 91.8% of which (19,739 genes) are supported by transcriptome data from leaves, roots and flowers. On average, protein-coding genes in *Z. marina* v.3.1 are 3,300 bp long and contain 4.99 exons, values that are very similar to those of *Z. marina* v.1.0. Notably, intron lengths greatly improved after manual curation ( Table 5). BUSCO assessment of the current gene set shows that the current annotation includes 95.7% complete genes in the embryophyte database10. 93.2% of the BUSCO genes were single copy while 2.5% of these BUSCO genes were found in duplicate. 0.5% of the BUSCO genes were fragmented and 3.8% was missing, which could be due to some specific pathways missing in *Z. marina*, compared to land plants ( Olsen et al., 2016) ( Table 6). BUSCO assessment in *Z. marina* v.3.1 shows more complete genes and fewer fragmented genes than for *Z. marina* v.1.0 ( Figure 1). 20,665 genes (96.2%) obtained at least one functional assignment based on similarity to known proteins in the databases. Pfam domain information could be added to 15,716 (73.2%) predicted genes, and 12,406 (57.7%) predicted genes could be assigned a GO term ( Table 7).

**Table 5.** Genome annotation statistics.

Statistics	*Zosma* v1.0	*Zosma* v3.1	*Zosma* v3.1_before_curation
Protein coding genes	20,450	21,483	21,533
Mean gene length, bp	3,301	3,300	3,202
Mean CDS length, bp	1,177	1,225	1,207
Mean exon per gene	5.20	5.00	4.9
Mean exon length, bp	226	245	245
Mean intron length, bp	443	710	510

**Table 6.** BUSCO completeness assessment of protein coding sequences in *Z. marina* version 3.1.

Total number of BUSCO core genes queried	1614
Number of core genes detected	
Complete	1544 (95.7%)
Complete + Partial	1552 (96.2%)
Number of missing core genes	62 (3.8%)
Scores in BUSCO format*	C:95.7% [S:93.2%, D:2.5%], F:0.5%, M:3.8%, n:1614

**Table 7.** Functional annotation.

Database	Count	Percentage
InterPro	17,073	79.5
PFAM	15,716	73.2
Gene3D	12,597	58.6
PANTHER	18,220	84.8
CDD	6,818	31.7
SUPERFAMILY	12,078	56.2
ProSite	8,969	41.7
GO	12,406	57.7
BLASTP	18,243	84.9
Swiss-Prot	14,374	66.9
Total annotation	20,665	96.2
Total unigene	21,483	100

## Conclusions

Here, we report a high-quality, chromosome-scale genome assembly of *Z. marina* using a combination of single-molecule real-time sequencing and Hi-C scaffolding. Although a draft genome sequence for *Z. marina* has been available for more than five years ( Olsen et al. 2016), a chromosome-scale assembly and well-annotated reference genome is an important step to further advance our understanding with respect to its metabolism, evolution and adaptation. As we expected, there is a discrepancy in genome size between an Illumina-derived assembly and a PACBIO long read-derived assembly, which is mainly due to the more accurate coverage of repetitive content. Nevertheless, the genome size of 259 Mb still characterizes *Z. marina* as relatively compact monocot genome, and it is also the smallest genome among the seagrasses where genome size estimates exist (JGI pilot analyses). Also, telomere and centromere regions are generally captured more fully. As a first high quality, reference genome of an important marine angiosperm, v.3.1 of the genome of *Z. marina* will provide a great resource for further comparative and evolutionary studies.

## Data availability

### Underlying data

**NCBI BioProject:***Zostera marina* Genome sequencing and assembly. Accession number: PRJNA701932; https://identifiers.org/ncbi/bioproject:PRJNA701932.

The genome assembly and annotation for *Z. marina* v.3.1 is also available from https://data.jgi.doe.gov/refine-download/phytozome?organism=Zmarina and through the ORCAE platform ( https://bioinformatics.psb.ugent.be/gdb/zostera/).
