# Supplementary material for: Improved chromosome-level genome assembly and annotation of the seagrass, Zostera marina (eelgrass)
Source: F1000Res. 2021 Apr 15;10:289. [Version 1] doi: 10.12688/f1000research.38156.1 (PMC8482049; doi:10.12688/f1000research.38156.1)
Supplement: Peer review history

# Peer review: Reviewer response for version 1

**DOI:** 10.5256/f1000research.41219.r89904

**Published:** 2021-09-29

**Authors:** Vratislav Peska

**Affiliations:** 1 Department of Cell Biology and Radiobiology, The Czech Academy of Sciences, Institute of Biophysics, Brno, Czech Republic

The manuscript "Improved chromosome-level genome assembly and annotation of the seagrass, *Zostera marina* (eelgrass)" by Ma, Olsen, Reusch *et al.* is a well-written article about a unique genome of marine angiosperm plant *Z. marina*. The results convincingly present improving the genome assembly using PacBio and Hi-C strategies. The improvement is obvious, among others, from an increased number of coding genes by 1000 in comparison with the first assembly and almost removing fragmented hits in the BUSCO analysis results.

 I must say that I do not have any serious concerns about the methodology, results, and conclusions. The release of an improved genome is a great opportunity of the research community to have a powerful tool in genome-dependent studies of this extraordinary plant.

 My minor notes:

1. In the text, "come online in the near future along with *Zostera mueller*" probably should be " *muelleri*".
2. The sentence, "The remaining scaffolds were screened against bacterial proteins, organelle sequences, GenBank nr and removed if found to be a contaminant." would need rewording to be clear what "GenBank nr" means. Does it mean anything that is not obviously of *Zostera* species origin?
3. I suggest using the "BWA-MEM algorithm" instead of "bwa mem".
4. I do not understand what the (“NNNNNNNNNNN”) stands for.
5. I think a short explanation of the assembly numbering would be helpful for readers. Was the Zosma2.0 published and is available somewhere? The JGI link shows v2.2 and v3.1. Actually v3.1 contains data named Zmarina_668_v2.0.fa.gz. The ugent link shows only V2. In summary, the numbering is confusing and a bit more intuitive naming would be nice.
6. In Peska *et al.,* (2020) ^1^, we showed there are two telomerase RNA genes in *Z. marina*. I wonder if assembly v3.1 confirms them as a set of separate genes or two alleles of the same gene? We also showed decompacted mitotic chromosome at the rDNA locus which might be a fragile site. Does it correspond to the observed "extra" pseudochromosome in the v3.1?

Is the work clearly and accurately presented and does it cite the current literature?

Yes

If applicable, is the statistical analysis and its interpretation appropriate?

Yes

Are all the source data underlying the results available to ensure full reproducibility?

Yes

Is the study design appropriate and is the work technically sound?

Yes

Are the conclusions drawn adequately supported by the results?

Yes

Are sufficient details of methods and analysis provided to allow replication by others?

Yes

Reviewer Expertise:

Telomere biology, genomics, sequencing

I confirm that I have read this submission and believe that I have an appropriate level of expertise to confirm that it is of an acceptable scientific standard.

## References

[1] : Human-like telomeres in Zostera marina reveal a mode of transition from the plant to the human telomeric sequences. *Journal of Experimental Botany*.2020;71(19) : 10.1093/jxb/eraa2935786-579310.1093/jxb/eraa29332589715

# Peer review: Reviewer response for version 1

**DOI:** 10.5256/f1000research.41219.r83351

**Published:** 2021-06-16

**Authors:** Jarkko Salojärvi

**Affiliations:** 1 School of Biological Sciences, Nanyang Technological University, Singapore, Singapore

The authors present a new assembly of eelgrass based on long read sequencing and Hi-C chromosome conformation capture technology. Altogether this is a well-prepared improvement on the existing genome assembly. In addition to improving the contiguity, also the manual curation of 1,460 gene models is very much welcome.

 For comparison purposes the authors could still produce a dot plot of the whole genome alignments of the old vs new version.

Is the work clearly and accurately presented and does it cite the current literature?

Yes

If applicable, is the statistical analysis and its interpretation appropriate?

Not applicable

Are all the source data underlying the results available to ensure full reproducibility?

Yes

Is the study design appropriate and is the work technically sound?

Yes

Are the conclusions drawn adequately supported by the results?

Yes

Are sufficient details of methods and analysis provided to allow replication by others?

Yes

Reviewer Expertise:

Genomics, population genetics, bioinformatics

I confirm that I have read this submission and believe that I have an appropriate level of expertise to confirm that it is of an acceptable scientific standard.
